# Antiviral treatment of COVID-19

**DOI:** 10.3906/sag-2004-145

**Published:** 2020-04-21

**Authors:** Serap ŞİMŞEK YAVUZ, Serhat ÜNAL

**Affiliations:** 1 Department of Infectious Disease and Clinical, Microbiology, İstanbul Faculty of Medicine, İstanbul University, İstanbul Turkey; 2 Department of Infectious Disease, Faculty of Medicine, Hacettepe University, Ankara Turkey

**Keywords:** Covid-19, Sars-CoV-2, antiviral

## Abstract

Currently, there is not any specific effective antiviral treatment for COVID-19. Although most of the COVID-19 patients have mild or moderate courses, up to 5%–10% can have severe, potentially life threatening course, there is an urgent need for effective drugs. Optimized supportive care remains the mainstay of therapy. There have been more than 300 clinical trials going on, various antiviral and immunomodulating agents are in various stages of evaluation for COVID-19 in those trials and some of them will be published in the next couple of months. Despite the urgent need to find an effective antiviral treatment for COVID-19 through randomized controlled studies, certain agents are being used all over the world based on either in-vitro or extrapolated evidence or observational studies. The most frequently used agents both in Turkey and all over the world including chloroquine, hydroxychloroquine, lopinavir/ritonavir, favipiravir and remdesivir will be reviewed here .Nitazoxanide and ivermectin were also included in this review as they have recently been reported to have an activity against SARS-CoV-2 in vitro and are licensed for the treatment of some other human infections.

## 1. Introduction

Currently, there is not any specific effective anti-viral treatment for COVID-19. Although most of the COVID-19 patients have mild or moderate course, up to 5-10% can have severe , potentially life threatening course, there is an urgent need for effective drugs [1]. Optimized supportive care remains the mainstay of therapy. As new data regarding clinical characteristics, treatment options, and outcomes for COVID-19 emerges approximately every hour, physicians who are in the care of patients should keep themselves up to date on this issue. There have been more than 300 clinical trials going on, and some of them will be published in the next couple of months. The WHO is launching “Solidarity” clinical trial for COVID-19 treatments to further evaluate remdesivir, hydroxychloroquine/chloroquine, and lopinavir-ritonavir with and without interferon beta^1^1World Health Organization (2020). “Solidarity” clinical trial for COVID-19 treatments [online]. Website https://www.who.int/ emergencies/diseases/novel-coronavirus-2019/global-research-on-novel-coronavirus-2019-ncov/solidarity-clinical-trial-for-covid-19-treatments [accessed 12 April 2020].. Various other antiviral and immunomodulating agents are in various stages of evaluation for COVID-19. A registry of international clinical trials can be found on the WHO website and at ClinicalTrials.gov.

At the moment, it is strongly recommended that patients be recruited into ongoing trials, which would provide much-needed evidence on the efficacy and safety of various therapies for COVID-19, given that we could not determine whether the benefits outweigh harms for most treatments [2]^2^2Bhimraj A, Morgan RL, Shumaker AH, Lavergne V, Baden5 L et al. Infectious DiseasesSociety of America Guidelines on the Treatment and Management of Patients with COVID-19 Infection [online]. Website https://www.idsociety.org/globalassets/idsa/practice-guidelines/covid-19/treatment/idsa-covid-19-gl-tx-and-mgmt-4-11-20-1058-am-edt.pdf [accessed 12 April 2020]..

Unless used in the context of randomized clinical trials, antivirals will not be proved to be efficacious or safe for the treatment of COVID-19. In the 2014 Ebola outbreak, close to 30, 000 individuals developed Ebola viral disease, and numerous therapies were tested against this virus, including chloroquine, hydroxychloroquine, favipiravir, brincidofovir, monoclonal antibodies, antisense RNA, and convalescent plasma, among many others. With such a large number of therapeutic interventions given to affected patients, the goal was to determine which was efficacious against Ebola. Ultimately, none proved to be efficacious or safe, just because of virtually all studies were single-group interventions without concurrent controls, which led to no definitive conclusion related to efficacy or safety. This tragedy of not discovering new therapies during an outbreak should not be repeated [3].

The vast majority of patients with COVID-19 will do fine without any therapy, so in most cases, there’s no need for antiviral therapy. However, waiting until patients are severely ill before initiating therapy could cause us to miss an early treatment window, during which the disease course is more modifiable. It is known that antiviral therapy is most likely to provide benefit when initiated earlier during the course of the disease both in influenza [4] and in SARS [5]. Predictors of adverse outcome might be useful in predicting who will do poorly and thus who might benefit most from early antiviral therapy^3^3Internet Book of Critical Care (IBCC) (2020). COVID-19 [online]. Website https://emcrit.org/ibcc/COVID19/#background_on_ antiviral_therapy [accessed 12 April 2020].. It is logical to start antiviral treatment as soon as possible also for COVID-19 patients especially in the case of the presence of predictors of adverse outcomes.

Combined usage of antiviral drugs for COVID-19 patients should be considered in the light of current knowledge and case by case; adverse drug reactions and drug-drug interactions should always be regarded.

Despite the urgent need to find an effective antiviral treatment for COVID-19 through randomized controlled studies, certain agents are being used all over the world based on either in vitro or extrapolated evidence or observational studies. The most frequently used agents both in Turkey and all over the world including chloroquine, hydroxychloroquine, lopinavir/ritonavir, favipiravir and remdesivir will be reviewed here^4^4T.C. Sağlık Bakanlığı Halk Sağlığı Genel Müdürlüğü (2020). COVID-19 (SARS-CoV-2 Enfeksiyonu Rehberi, Bilim Kurulu Çalışması [online]. Website https://covid19bilgi.saglik.gov.tr/depo/rehberler/COVID-19_Rehberi.pdf [accessed 12 April 2020].^5^5Institute of Tropical Medicine, Universiteit Antwerten, CHU Saint-Pierre, Sciensano, AFMPS Fagg (2020). Interim clinical guidance for adults with suspected or confirmed covid-19 in Belgium, Version 7 [online]. Website https://epidemio.wiv-isp.be/ID/Documents/ Covid19/COVID-19_InterimGuidelines_Treatment_ENG.pdf [accessed 12 April 2020].^6^6World Health Organization (2020). Report of the WHO-China joint mission on coronavirus disease 2019 (COVID-19) [online]. Website https://www.who.int/docs/default-source/coronaviruse/who-china-joint-mission-on-covid-19-final-report.pdf [accessed 12 April 2020].^7^7Nebraska Medicine (2020). COVID-19 Antiviral and pharmacotherapy recommendations [online]. Website https://www.nebraskamed. com/sites/default/files/documents/covid-19/covid19-antiviral-pharmacotherapy-recommendations.pdf [accessed 12 April 2020].. Nitazoxanide and ivermectin were also included in this review as they have recently been reported to have an activity against SARS-CoV-2 in vitro and are licensed for the treatment of some other human infections. Mechanisms of actions of these drugs were shown in Table.

**Table 1:** Antivirals investigated for the treatment of COVID-19 in clinical trials or in vitro studies.

Group	Drugs	Mechanism of action	Dosing
Inhibitors of viral RNA polymerase /RNA synthesis	Remdesivir (GS-5734)	Adenosine nucleotide analogue, prodrug, RdRp inhibitor	Day 1: 200mg, IVDay 2–5 (or 10): 100 mg/day, IV
	Favipiravir	Guanosinenucleotid analogue, prodrug, RdRp inhibitor	Day 1: 2X1600 mgDay 2–7 (or 10): 2 × 600 mg/day
Inhibitors of viral protein synthesis	Lopinavir/ritonavir	Protease inhibitor	Day 1–10 (or14): 400mg/100mg × 2/day, orally
Viral entry inhibitors	Hydroxychloroquine	Increasing endosomal pH required for virus/cell fusion, as well as interfering with the glycosylation of cellular receptors of SARS-CoV (ACE-2)	Day 1–5: 2 × 200 mg/day, orally
	Chloroquine		Day 1–5 (or 10): 2 × 500 mg/day, orally
Imunomodulators	Nitazoxanide	Interference with host-regulated pathways involved in viral replication, amplifying cytoplasmic RNA sensing and type I IFN pathways	
	Ivermectin	Inhibition nuclear import of host and viral proteins through inhibition of importin 1 heterodimer	

Modified from “Hoffmann C. Treatment. In: Kamps BS, Hoffmann C, eds. Covid Reference, Edition 2020-2. Website www.covidreference. com”

## 1.1. Hydroxychloroquine and Chloroquine 

Chloroquine (CQ) and hydroxychloroquine (HCQ) are aminoquinolines, which have been used to treat malaria and autoimmune diseases for over 50 years. Besides their antimalarial effects, these two drugs possess immunomodulatory effects allowing them to use for the treatment of autoimmune conditions such as systemic lupus erythematosus and rheumatoid arthritis. Hydroxychloroquine and chloroquine can inhibit certain cellular functions and molecular pathways involved in immune activation [Inhibition of MHC class II expression, antigen presentation and immune activation (reducing CD154 expression by T cells); inhibition of production of various proinflammatory cytokines, such as IL-1, IFNα and TNF, which can protect against cytokine-mediated cartilage resorption; interference with Toll-like receptor 7 (TLR7) and TLR9 signaling pathways; interference with cyclic GMP-AMP (cGAMP) synthase (cGAS) activity] partly by accumulating in lysosomes and auto phagosomes of phagocytic cells and changing local pH concentrations [6]. Chloroquine analogs are weak diprotic bases (can accept more two protons) and they can penetrate and concentrate within acidic organelles such as endosomes and lysosomes which leads to elevated intra-vesicular pH resulting in prevention of endosome trafficking and prevents viral fusion into the cell. This mechanism has translated to the potential role of these drugs in the treatment of COVID-19. Additionally, studies also revealed that these drugs interfers with the glycosylation of ACE-2 receptor which prevents SARS-CoV-2 receptor binding and subsequent infection. Recent in vitro studies reported CQ and HCQ effective against SARS-CoV-2 at a multiplicity of infection (MOI) of 0.01 with a 50% effective concentration (EC50) of 2.71 µM and 4.51 µM in Vero E6 cells, respectively. At all MOIs (0.01, 0.02, 0.2, and 0.8), EC50 for CQ (2.71, 3.81, 7.14, and 7.36 μM) was lower than that of HCQ (4.51, 4.06, 17.31, and 12.96 μM) [7–9]. In another in vitro analysis, HCQ was found to be more potent than CQ at inhibiting SARS-CoV-2 and HCQ sulfate 400 mg given twice daily for 1 day, followed by 200 mg twice daily for 4 more days is recommended to treat SARS-CoV-2 infection [10,11]. 

The use of CQ or HCQ is included in COVID-19 treatment guidelines all over the world but data supporting this is quite scare. An early report from China suggested that chloroquine usage was associated with reduced progression of the disease and decreased duration of symptoms [12]. However, primary data supporting these claims have not been published. 

In a prospective randomized trial of 30 adults with COVID-19 in China, 15 patients treated with 400 mg HCQ + conventional treatment were compared with 15 patients treated with conventional treatment only. The proportion of patients with nasopharyngeal viral clearance at day 7, mean viral clearance time, temperature normalization and progression rate in CT  

were not found to be different between the groups, and one patient in the HCQ group progressed to severe disease [13] .

In another randomized trial of 62 patients with mild COVID-19 pneumonia without hypoxia reported that the body temperature recovery time and the cough remission time were significantly shortened in the HCQ treatment group. Additionally, HCQ treatment group experienced more improvement of pneumonia symptoms (80.6%, 25 of 31) compared with the control group (54.8%, 17 of 31). Notably, all 4 patients progressed to severe illness occurred in the control group. However, there were 2 patients with mild adverse reactions in the HCQ treatment group. But, the trial has not been published in a peer-reviewed journal, and there are concerns about concomitant co-therapies, baseline differences between the groups, and lack of blinding or placebo control^8^8Chen Z, Hu J, Zhang Z, Jiang S, Han S et al. (2020). Efficacy of hydrochlroquine in patients with COVID-19: Results of a randomized trial [online]. Website https://www.medrxiv.org/content/10.1101/2020.03.22.20040758v2 [accessed 12 April 2020]..

Other published clinical data on either of these agents are limited and have methodologic problems. In an open-label case-control study of 36 adults with COVID-19, use of HCQ (200 mg three times per day for 10 days) was associated with a higher rate of undetectable SARS-CoV-2 RNA on nasopharyngeal specimens at day 6 compared with no specific treatment (70 versus 12.5 %, (P < 0.001). While the combination of HCQ with azithromycin resulted in 100% viral clearance in 6 patients, HCQ alone resulted in %57 clearance in 14 patients (P < 0.001) [14]. 

The same group also published the results of 80 COVID-19 patients receiving a combination of HCQ and azithromycin. They noted clinical improvement in all but one 86-year-old patient who died and one 74-year-old patient still in the intensive care unit. A rapid fall of nasopharyngeal viral load tested by qPCR was noted, with 83% negative at day 7, and 93% on day 8. Virus cultures from patient’s respiratory samples were negative in 97.5% patients on day5 [15]. Both of these studies have substantial methodological problems that cast doubt on the conclusions [2]^9^9International Society of Antimicrobial Chemotherapy (2020). Statement on IJAA paper[online]. Website https://www.isac.world/ news-and-publications/official-isac-statement [accessed 12 April 2020]..

Unfortunately, these studies have resulted in the massive adoption of the regimen by clinicians worldwide. There are also increasing concerns about the safety of these drugs: both medications have been independently shown to increase the risk for QT interval prolongation, drug-induced torsade’s de pointes, and drug induced-sudden cardiac death [16]^10^10Chang R, Sun WZ (2020). Repositioning chloroquine as an ideal antiviral prophylaxis against COVID-19-time is now [online]. Website https://www.preprints.org/manuscript/202003.0279/v1 . [accessed 12 April 2020]..

In a preliminary safety result of a randomized, double-blinded, phase IIb clinical trial (CloroCovid-19 Study) aiming to assess safety and efficacy of two different CQ dosages as adjunctive therapy of hospitalized patients with SARS-CoV-2, the high dose CQ arm presented more QTc > 500 ms (25%), and a trend toward higher lethality (17%) than the lower dosage in the first recruited 81 patients. Preliminary findings suggest that the higher CQ dosage (10-day regimen) should not be recommended for COVID-19 treatment because of its potential safety hazards. As a result, investigators prematurely halted patient recruitment to this arm** [**17]. 

In another recent study from USA, researchers reported the change in the QT interval in 84 adult patients with SARS-CoV-2 infection treated with HCQ/Azithromycin combination. They reported that QTc prolonged maximally from baseline between days 3 and 4, in 30% of patients QTc increased by greater than 40 ms and in 11% of patients QTc increased to >500 ms, representing high-risk group for arrhythmia. The development of acute renal failure but not baseline QTc was a strong predictor of extreme QTc prolongation.^11^11Borba MGS, De Almeida Val F, Sampaio VS, Araújo Alexandre MA, Melo GC et al. (2020). Chloroquine diphosphate in two different dosages as adjunctive therapy ofhospitalized patients with severe respiratory syndrome in the context of coronavirus (SARS-CoV-2) infection: preliminary safety results of a randomized, double-blinded, phase IIb clinical trial (CloroCovid-19 Study) [online]. Website https://www.medrxiv.org/content/10.1101/2020.04.07.20056424v2 [accessed 12 April 2020].

Finally, a total of 54 serious cardiac events, 7 sudden cardiac arrests (4 deaths), 37 prolonged QT and 10 arytmia + syncope have been reported to French National Pharmacovigilance Agency since the 27th of March 2020^12^12Chorin E, Dai M, Shulman E, Wadhwani L, Cohen RB et al. (2020). The QT interval in patients with SARS-CoV-2 infection treated with hydroxychloroquine/azithromycin [online]. Website https://www.medrxiv.org/content/10.1101/2020.04.02.20047050v1 [accessed 12 April 2020]..

CQ and HCQ have also been suggested as a candidate for antiviral prophylaxis against the current COVID-19 pandemic because of its demonstrated mechanisms of action of preventing viral entry and fusion, evidence of in vitro efficacy at a clinically achievable dose and high tissue concentration as well as preliminary clinical evidence of efficacy as a treatment. But there is also insufficient data to support this suggestion and these agents should not be used as prophylactic agents for SARS-CoV-2 except in the context of a clinical trial^13^13Le Monde (2020). Covid-19-les-effets-indesirables-graves-s-accumulent-sur-l-hydroxychloroquine [online]. Website https://www.lemonde.fr/planete/article/2020/04/09/covid-19-les-effets-indesirables-graves-s-accumulent-sur-lhydroxychloroquine_ 6036139_3244.html [accessed 12 April 2020]..

There are insufficient data thus far to know whether HCQ or CQ has a role either in the treatment or in the prophylaxis of COVID-19. Beside antimalarial drugs can cause ventricular arrhythmias, QT prolongation, and other cardiac toxicity, which may pose a particular risk to critically ill persons. Given these serious potential adverse effects, the hasty and inappropriate interpretation of the literature by public leaders has the potential to do serious harm. For these reasons, it is strongly recommended that patients should be referred to a clinical trial whenever possible. Ongoing trials for HCQ are actively recruiting with hopes to further delineate its role in the treatment and prophylaxis of COVID-19 [11]. 

## 1.2. Favipiravir  

Favipiravir (T-705; 6-fluoro-3-hydroxy-2-pyrazinecarboxamide) is an antiviral agent that selectively and potently inhibits the RNA-dependent RNA polymerase (RdRp) of RNA viruses. Favipiravir undergoes an intracellular phosphoribosylation to be an active form, favipiravir ribofuranosyl-5B-triphosphate (favipiravir-RTP), which is recognized as a substrate by RdRp, and inhibits the RNA polymerase activity. Since the catalytic domain of RdRp is conserved among various types of RNA viruses, this mechanism of action may underpins a broader spectrum of antiviral activities of favipiravir. Favipiravir-RTP inhibits RdRp of the influenza virus with an IC50 of0.022 µg/mL, but does not affect the human DNA polymerases α, β, γ subunits at up to 100 µg/mL. In addition to the inhibition of influenza virus, favipiravir shows inhibitory effects on a wide range of RNA viruses, such as arena-, bunya-, flavi- and filoviruses causing hemorrhagic fevers [18, 19]. It has been shown to be effective in the treatment of influenza and in some extent Ebola virus disease [19–21]. Genome sequencing of the 2019-nCoV identified the virus as a single-stranded RNA beta-coronavirus with the RdRp gene similar to those of SARS-CoV and MERS-CoV. Therefore, favipiravir is considered as one of the potential candidates for COVID-19, though confirmed in vitro and preclinical animal studies are not available yet. In an in-vitro study, SARS-CoV-2 was inhibited by favipiravir in Vero E6 cells with an EC50 of 61.88 µMol [14]. But in another study favipiravir showed no apparent antiviral effect against the SARS-CoV-2 virus in vitro at concentrations under 100 μML [22].

In an open-label, controlled study of 80 patients with laboratory-confirmed COVID-19, 35 patients who received oral favipravir plus interferon (IFN)-α by aerosol inhalation were compared with 45 patients who received lopinavir/ritonavir plus IFN-α by aerosol inhalation. All baseline characteristics were comparable between the two arms. A shorter viral clearance time was found for the favipiravir arm versus the control arm [median (interquartile range, IQR), 4 (2.5–9) d versus 11 (8–13) d, P < 0.001]. The favipiravir arm also showed significant improvement in chest imaging compared with the control arm, with an improvement rate of 91.43% versus 62.22% (P = 0.004). After adjustment for potential confounders, the favipiravir arm also showed a significantly higher improvement rate in chest imaging. Multivariable Cox regression showed that favipiravir was independently associated with faster viral clearance [23]. But this article has been temporarily removed by the publisher and the reason for the removal of the article has not been specified yet and rising the suspicion on the results of the study.  

In a randomized clinical trial^14^14Chen C, Huang J, Cheng Z, Zhang Y, Cheng Z et al. (2020). Favipiravir versus arbidol for COVID-19: a randomized clinical trial [online]. Website https://www.medrxiv.org/content/medrxiv/early/2020/04/08/2020.03.17.20037432.full.pdf [accessed 12 April 2020]. 120 patients who were assigned to the favipiravir group compared with 120 arbidol treated patients. In patients with mild-moderate COVID-19, 7 day’s clinical recovery rate was 55.86% in the arbidol group and 71.43% in the favipiravir group (P = 0.0199). For mild-moderate COVID-19 patients the time of fever reduction and cough relief in the favipiravir group was significantly shorter than that in the arbidol group (both P < 0.001), no difference was observed of auxiliary oxygen therapy or noninvasive mechanical ventilation rate (both P > 0.05). The most possible adverse events were abnormal liver function tests, psychiatric symptom reactions, digestive tract reactions and raised serum uric acid [3 (2.50 %) in arbidol group vs. 16 (13.79%) in favipiravir group, P < 0.0001]. These trials have not been published in a peer-reviewed journal, and there are concerns about concomitant co-therapies, baseline differences between the groups, and lack of blinding or placebo control. 

At the moment current knowledge is not enough to recommend favipiravir for the treatment of COVID-19 and additional studies are needed. There are several RCT going on in China. 

## 1.3. Remdesivir

Remdesivir is a novel antiviral drug developed by Gilead Sciences, originally for the treatment of Ebola virus disease and Marburg virus infections. Remdesivir is a prodrug of a nucleotide analog that is intracellularly metabolized to an analog of adenosine triphosphate that inhibits viral RNA polymerases. Remdesivir has broadspectrum activity against members of several virus families, including filoviruses (e.g., Ebola) and coronaviruses [e.g., SARS-CoV and Middle East respiratory syndrome coronavirus (MERSCoV)] and has shown prophylactic and therapeutic efficacy in nonclinical models of these coronaviruses. In vitro testing has also shown that remdesivir has activity against SARS-CoV-2 with an EC50 value of 1.76 μM in Vero E6 cells suggesting its working concentration is likely to be achieved in nonhuman primate models [8]. Treatment with intravenous remdesivir showed significant improvement for the first COVID-19 case in US [24] and then a trial has been initiated quickly to assess the efficacy and safety of remdesivir in patients hospitalized with 2019-nCoV infection. In a cohort of patients hospitalized for severe Covid-19 who were treated with compassionate use remdesivir, clinical improvement was observed in 36 of 53 patients (68%) [25]. As there was no placebo or active comparator in this study, it is hard to draw any concrete conclusions and measurement of efficacy will require results of ongoing randomized, placebo-controlled trials of remdesivir therapy. There are 4 clinical trials currently enrolling patients in the United States and, two additional trials recruiting only in China have been registered on ClinicalTrials.gov, NCT04257656 (severe disease) and NCT04252664 (mild-moderate disease) [2].

## 1.4. Lopinavir/ritonavir( LPV/r) 

Lopinavir is a protease inhibitor used to treat HIV infection, with ritonavir as a booster. Protease is a key enzyme in coronavirus polyprotein processing and lopinavir and/or ritonavir has anti coronavirus activity in vitro. Most in vitro studies have shown that SARS‐CoV could be inhibited by lopinavir and that the EC50 of lopinavir is acceptable. Lopinavir showed an antiviral effect against SARS-CoV-2 virus in Vero E6 cells with the estimated EC50 at 26.63 μM [26].

Furthermore, two retrospective matched cohort studies of SARS patients revealed that LPV/r plays an essential role in the clinical outcome, especially in the early stage. In a study from Hong-Kong, compared with ribavirin alone, patients treated with lopinavir/ritonavir plus ribavirin had a lower risk of acute respiratory distress syndrome (ARDS) or death caused by SARS-CoV, 2.4% vs. 28.8%, P = 0.001) at day 21 after the onset of symptoms [27]. 

LPV/r treatment alone or in combination with interferon had improved clinical outcomes in experiments involving common marmosets and in some MERS patient [28].

Postexposure prophylaxis with LPV/r was found to be associated with a 40% decrease in the risk of MERS infection, although the design of the study was raised some concerns [29]. 

Five patients with COVID-19 in Singapore were treated with LPV/r within 1 to 3 days of desaturation, but evidence of clinical benefit was equivocal. While defervescence occurred within 1 to 3 days of LPV/r initiation, it was unable to prevent progressive disease in 2 patients. A decline in viral load as indicated by the cycle threshold value from nasopharyngeal swabs also appeared similar between those treated and not treated with LPV/r [30].

In another study of of 47 patients with COVID-19; compared with the standard of care (arbidol plus IFN-α inhaler) (SOC) (5 patients), the combination treatment with LPV/r plus SOC (42 patients) has resulted in a shorter time (test group: 4.8 ± 1.94 days vs. control group: 7.3 ± 1.53 days, P = 0.0364) to return normal body temperature and to be negative for SARS-CoV-2 test in clinical samples (7.8 ± 3.09 days vs. 12.0 ± 0.82 days, P = 0.0219) [31].

In another study, 44 patients with mild/moderate COVID-19 were randomly assigned to receive LPV/r (21 patients), arbidol(16 patients) and no antiviral medication as control (7 patients). No statistical differences were found among three groups in the rates of antipyretics, cough alleviation, improvement of chest CT or the deterioration rate of clinical status (all P > 0.05). Overall, 5 (23.8%) patients in the LPV/r group experienced adverse events during the follow-up period. No apparent adverse events occurred in the arbidol or control group. It was concluded from this study that LPV/r or arbidol monotherapy seems little benefit for improving the clinical outcome of mild/moderate COVID-19 and LPV/r might lead to more adverse events^15^15Li Y, Xie Z, Lin W, Cai W, Wen C, et al. An exploratory randomized controlled study on the efficacy and safety of lopinavir/ritonavir or arbidol treating adult patients hospitalized with mild/moderate COVID-19 (ELACOI). [online]. Website https://www.medrxiv.org/ content/10.1101/2020.03.19.20038984v2 [accessed 12 April 2020]. .

In a randomized trial of 199 patients with severe COVID-19, the addition of LPV/r (400/100 mg) twice daily for 14 days to standard care did not decrease the time to clinical improvement compared with standard care alone [32]. There was a trend towards decreased mortality with LPV/r (19 versus 25 percent), and the numerical difference in mortality was greater among those who were randomized within 12 days of symptom onset, but neither difference was statistically significant. The rate of SARS-CoV-2 decline was similar in the group that received LPV/r and the group that did not. LPV/r was stopped early in 14 percent because of adverse effects. The patients recruited for the study were late in infection and already had considerable tissue damage (as evidenced by compromised lung function and 25% mortality in the control group). Even highly active antibacterial agents have limited efficacy in advanced bacterial pneumonia [33]. Accelerated clinical recovery (16.0 days vs. 17.0 days) and reduced mortality (19.0% vs. 27.1%) were observed in a post hoc subgroup of those treated within 12 days after the onset of symptoms, but not in those treated later [48, 49]. Also in another study of 280 COVID-19 patients, time from illness onset to antiviral was found to be a risk factor for severe disease. Patients in the mild group experienced earlier initiation of antiviral treatment (1.19 ± 0.45 vs. 2.65 ± 1.06 days in the severe group, P < 0.001) [34]. The question of whether earlier LPV/r treatment in COVID-19 could have clinical benefit is an important one that requires further studies. 

With the available data, it is difficult to assess whether LPV/r has a role for the treatment of COVID-19 either as monotherapy or in combination, limited data suggesting no advantage over standard care for SARS-CoV-2. Importantly, it warrants comment that in the recent randomized controlled trial in COVID-19 pneumonia the median time from symptom onset to initiation of therapy was 13 days, and in the SARS CoV-1 experience therapy appeared effective if started early, but not as rescue/salvage. 

If utilized, drug interactions must be screened and gastrointestinal toxicities, including diarrhea, nausea, and vomiting, and hepatoxicity require close monitoring, particularly since elevated AST or ALT may exclude patients with COVID-19 from clinical trials. 

If LPV/r is used, the patient’s HIV status should be known and if the patient has HIV, LPV/r should be used as part of a standard combination antiretroviral regimen.

## 1.5. Nitazoxanide

Nitazoxanide and its active metabolite, tizoxanide have demonstrated potent in vitro activity against SARS CoV-2 and MERS CoV in Vero E6 cells with an EC50 of 2.12 μM and 0.92 μM, respectively. It also displays broad-spectrum in vitro antiviral activity

against influenza, respiratory syncytial virus, parainfluenza, rotavirus, and norovirus among others in addition to coronaviruses. This broad-spectrum antiviral activity is believed to be due to the fact that the mechanism of action is based on interference with host-regulated pathways involved in viral replication rather than virus-specific pathways [35]. Nitazoxanideupregulates the innate antiviral mechanisms by broadly amplifying cytoplasmic RNA sensing and type I IFN pathways. Nitazoxanide interferes with the viral infection by upregulating the precise host mechanisms that viruses target to bypass host cellular defenses [36]. Due to its broad-spectrum antiviral activity, nitazoxanide is being investigated in clinical trials including randomized controlled ones for the management of influenza and other acute respiratory infections, although results are not encouraging or unavailable yet. Although the in vitro activity of nitazoxanide against SARS-CoV-2 is encouraging, more data are clearly needed to determine its role in the management of COVID-19 [26]^16^16Padmanabhan S (2020). Potential dual therapeutic approach against SARS-CoV-2/COVID-19 with Nitazoxanide and Hydroxychloroquine [online].Website https://www.researchgate.net/profile/Srivatsan_Padmanabhan/publication/339941717_Potential_dual_therapeutic_approach_against_SARS-CoV-2COVID-19_with_Nitazoxanide_and_Hydroxychloroquine/links/5e825a6aa6fdcc139c173bc2/Potential-dual-therapeutic-approach-against-SARS-CoV-2-COVID-19-with-Nitazoxanide-and-Hydroxychloroquine.pdf. [accessed 12 April 2020]..

## 1.6. Ivermectin

Ivermectin is an FDA-approved broad-spectrum antiparasitic agent that in recent years, it has shown to have antiviral activity against a broad range of viruses in vitro. Originally identified as an inhibitor of the interaction between the human immunodeficiency virus-1 (HIV-1) integraseprotein and the importin (IMP) 1 heterodimer responsible for integrase protein nuclear import, ivermectin has since been confirmed to inhibit integrase protein nuclear import and HIV-1 replication. Other actions of ivermectin have been reported, but ivermectin has been shown to inhibit the nuclear import of host and viral proteins. It has been demonstrated to limit infection by some RNA viruses including influenza, dengue and West Nile viruses. Ivermectin has similarly been shown to be effective against the DNA virus pseudorabies virus (PRV) both in vitro and in vivo, with ivermectin treatment shown to increase survival in PRV-infected mice [37]. Efficacy was not observed for ivermectin against Zikavirus in mice, but the authors acknowledged that study limitations justified the reevaluation of ivermectin’s anti-Zika virus activity [38]. Finally, ivermectin was the focus of a phase III clinical trial in Thailand in 2014–2017, against dengue virus infection, in which a single daily oral dose was observed to be safe and resulted in a significant reduction in serum levels of viral NS1 protein, but no change in viremia or clinical benefit was observed^17^17Yamasmith E, Avirutnan P, Mairiang D, Tanrumluk S, Suputtamongkol Y et al. (2018). Efficacy and safety of ivermectin against dengue infection: a Phase III, randomized, double-blind, placebo-controlled trial [online]. Website http://www.rcpt.org/abstractdb/media/abstract/CON2018/Best%20Resident27/BRA_77_Eakkawit.pdf [accessed 12 April 2020].. 

In an in vitro study, ivermectin was found to be an inhibitor of the SARS-CoV-2, with a single addition to Vero-hSLAM cells 2 h post infection with SARS-CoV-2 able to effect ~5000-fold reduction in viral RNA at 48 h. Authors hypothesize that this was likely through inhibiting IMPα/β1- mediated nuclear import of viral proteins (as shown for other RNA viruses) and this inhibition disrupts the immune evasion mechanism of virus [38]. Further in vitro, in vivo and clinical trials are needed to determine its role in the management of COVID-19.
